# Infective endocarditis complicated by embolic events: Pathogenesis and predictors

**DOI:** 10.1002/clc.23554

**Published:** 2021-02-01

**Authors:** Wangling Hu, Xindi Wang, Guanhua Su

**Affiliations:** ^1^ Department of Cardiology Union Hospital, Tongji Medical College, Huazhong University of Science and Technology Wuhan China; ^2^ Department of Hematology Union Hospital, Tongji Medical College, Huazhong University of Science and Technology Wuhan China

**Keywords:** embolism, infective endocarditis, pathogenesis, predictors

## Abstract

**Background:**

Infective endocarditis (IE) continues to be associated with great challenges. Embolic events (EE) are frequent and life‐threatening complications in IE patients. It remains challenging to predict and assess the embolic risk in individual patients with IE accurately.

**Hypothesis:**

Accurate prediction of embolization is critical in the early identification and treatment of risky and potentially embolic lesions in patients with IE.

**Methods:**

We searched the PubMed, Web of Science, and Google Scholar databases using a range of related search terms, and reviewed the literatures about the pathogenesis and embolic predictors of IE.

**Results:**

The development of IE and its complications is widely accepted as the result of complex interactions between microorganisms, valve endothelium, and host immune responses. The predictive value of echocardiographic characteristics is the most powerful for EE. In addition, both easily obtained blood biomarkers such as C‐reactive protein, mean platelet volume, neutrophil‐to‐lymphocyte ratio, anti‐β2‐glycoprotein I antibodies, D‐Dimer, troponin I, matrix metalloproteinases, and several microbiological or clinical characteristics might be promising as potential predictors of EE.

**Conclusion:**

Our review provides a synthesis of current knowledge regarding the pathogenesis and predictors of embolism in IE along with a review of potentially emerging biomarkers.

## 1 INTRODUCTION

Infective endocarditis (IE) continues to be associated with great challenges. The IE incidence is approximately 3–10 per 100 000 people, with a high mortality rate of up to 30% at 30 days.^1^ The population at risk is increasing due in part to healthcare‐associated IE, which now accounts for 25%–30% of newly reported cases.^1^ With the epidemic of intravenous (IV) drug abuse, the incidence of community‐acquired IE is higher than expected.^2^ Embolic events (EE) are frequent and life‐threatening complications in IE, and up to 25% of IE patients exhibit EE at the time of diagnosis.^3^ Patients with high embolic risk (ER) should be identified early to allow for appropriate decisions related to EE management and prevention. However, it remains challenging to predict and assess the ER in individual patients accurately.

The pathogenesis of IE is complex, and it is still unclear why certain bacteria can adhere, survive, and cause damage in such a seemingly unfavorable environment as the cardiac valves. Insufficient understanding of the specific underlying mechanisms involved in the pathogenesis of IE is one of the barriers to improving the management of IE and associated EE.

In this review, we focused on the pathophysiology of IE and potential predictors of embolic complications.

## 2 METHODS

We searched the PubMed, Web of Science, and Google Scholar databases using a range of search terms, including “endocarditis,” “emboli,” “mechanism,” “pathogenesis,” “predict*,” “risk factor,” together with “right” or “left,” “prosthetic,” or “native.” We reviewed journals published in English primarily from the past 20 years. However, the search also included highly regarded older publications. We searched the reference lists of the articles identified using this search strategy and selected additional articles that were used to explain some concepts in more detail.

## 3 EMBOLISM COMPLICATIONS ASSOCIATED WITH IE

Currently, the imaging methods used to assess EE primarily include ultrasound, MRI, CT, and PET‐CT. The brain is the most common site of embolization, followed by solid organs, including the spleen, kidney, and lung. Less common sites of embolization included peripheral arteries, coronary circulation, and eyes.^4^

Appropriate antimicrobial therapy remains the preferred treatment to prevent EE.^3^ However, there is no evidence to suggest that prolonged antimicrobial treatment can effectively reduce the incidence of EE. Guidelines recommend that the selection of antibiotics should be based on the sensitivity of the newly isolated bacteria, and the duration of antibacterial treatment is usually 2–6 weeks.^3^

Patients with IE might also benefit from early surgery during the first several days following the initiation of antibiotic therapy because the risk of EE is highest at that time. According to the 2015 ESC guidelines, the primary indications for the use of surgery to prevent EE are the presence of persistent vegetations >10 mm and one or more previous episodes of EE despite appropriate antibiotic therapy.^3^

## 4 PATHOGENESIS

Decades of research have provided valuable information concerning the underlying pathogenesis of IE, although the mechanisms by which pathogens cause IE have not been fully elucidated. Currently, the development of IE and its complications is widely accepted as the result of complex interactions between microorganisms, valve endothelium, and host immune responses.

### 4.1 Vegetation and bacteremia

Frequently, IE is initiated by an endothelial injury that results in exposure of the subendothelial extracellular matrix that activates platelets and causes the formation of a fibrin‐platelet clot.^5^ The sterile cardiac lesion consists primarily of platelets and fibrin and is called non‐bacterial thrombotic endocarditis (NBTE). The pathogenesis of NBTE involves injured or inflamed endothelium, combined with a hypercoagulable state.^6^ Because the vegetations in NBTE are friable, systemic EE occurs more frequently in NBTE than in IE. Trousseau's syndrome has been reported as being associated with endocarditis, and NBTE can be the first presentation that leads to the diagnosis of Trousseau's syndrome.

Subsequently, microorganisms in the blood adhere to the fibrin‐platelet clot to initiate vegetation formation. Pathogenesis also includes critical components of innate immunity and pattern‐recognition receptors on the surfaces of epithelial cells, leukocytes, and platelets that recognize the bacteria.^5^ These factors result in an invasive infection resulting in the subsequent release of various cytokines.^5^ The activated epithelial cells transform their original anticoagulant state into a pro‐coagulant state. Tissue factors (TF) are released by activated epithelial cells and white blood cells. Meanwhile, dead cells and bacterial cell wall complexes are associated with the activation of factor XII. These factors facilitate activation of the extrinsic and intrinsic coagulation pathways, respectively.

### 4.2 Activation of coagulation and the complement and innate immune system

Coagulation has been proposed to be “at the heart” of IE.^5^ The coagulation system activates the complement system in several different ways.^7^ Thrombin, the cleavage product that forms as a result of the coagulation cascade, plays a fundamental role in hemostasis and bridges clotting and inflammation. The strong association between the coagulation and the innate immune system, which is referred to as immunothrombosis,^5^ is maintained in a delicate balance in healthy people. When this balance is disrupted, a range of diseases, especially IE, can occur.

### 4.3 Factors responsible for the valve's susceptibility to infection

**FIGURE 1:**
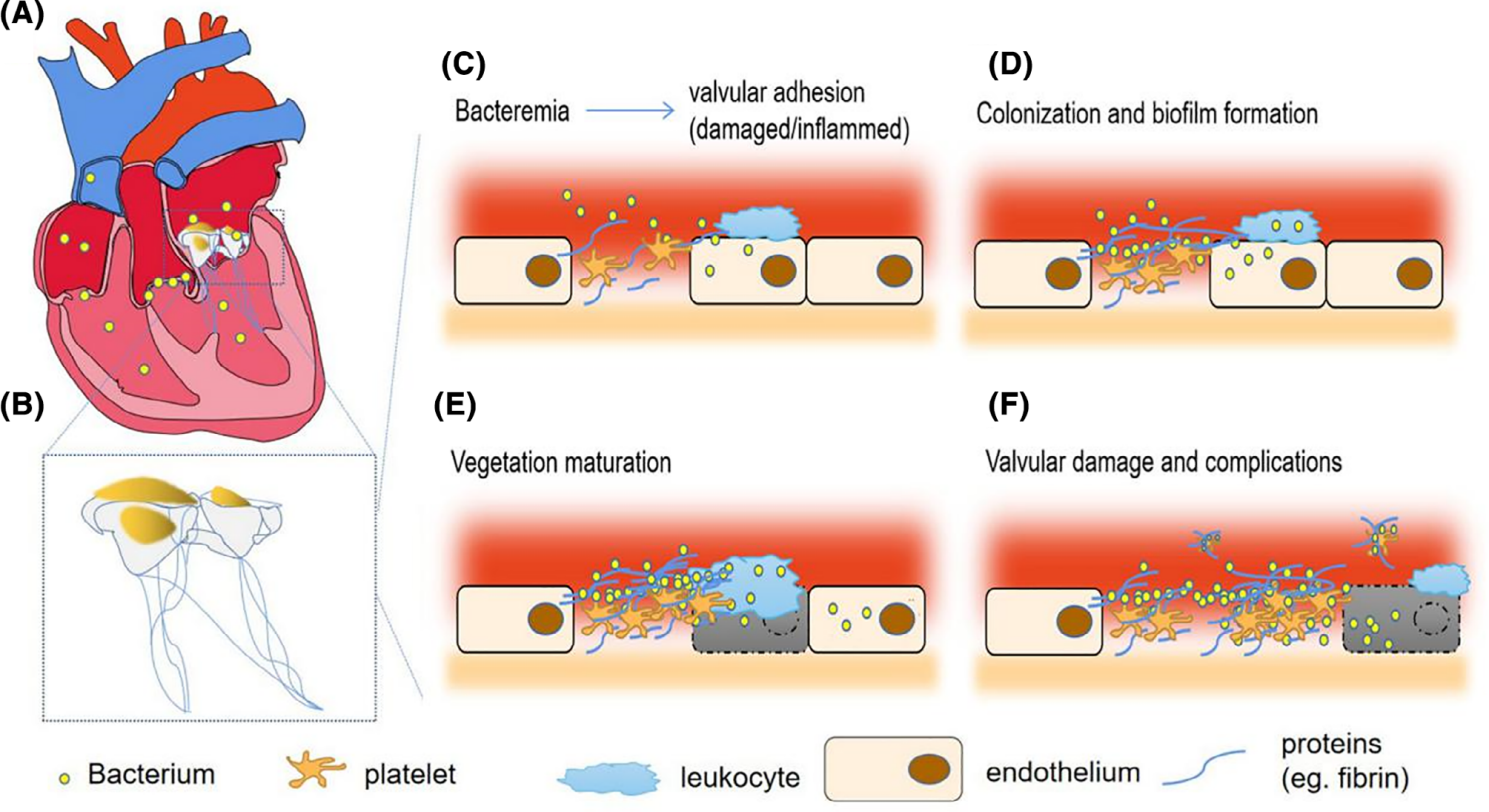
Pathogenesis of infective endocarditis (IE) and its complications. (A,B) Mitral valve vegetations in infectious endocarditis. (C) Bacteria in the bloodstream adhere to damaged or inflamed valves and cause invasive infection. (D) Bacterial persistence and proliferation lead to valve colonization and biofilm formation (especially *Staphylococcus aureus*). (E) The valvular lesion progresses further, followed by vegetation maturation. (F) Severe valvular damage and disseminated vegetation particles result in clinical symptoms and various complications

Tissue destruction and inflammation are responsible for the valve's susceptibility to infection. Mechanical damage or inflammation of the cardiac endothelium, combined with an enhanced coagulation state and an imbalanced innate immune system, leads to platelet‐fibrin aggregates in an environment that proves suitable for deposition. The deposition of these aggregates makes the valve more vulnerable to pathogens **(**Figure 1).

### 4.4 Valve colonization

Bacterial adhesion to damaged or inflamed valves is the initial step in valve colonization and ensuing vegetation formation and maturation. In this process, most endocarditis pathogens need to overcome the strong shear forces present in the chambers of the heart by binding and activating platelets and using them like bridges.^8^ In addition to platelets, *Staphylococcus aureus* produces von Willebrand factor‐binding protein (vWFbp) that binds to the A1 domain of vWF directly and expresses two types of fibronectin‐binding proteins (FnBPs), which allows the bacteria to interact with fibronectin.^9^ For *S. aureus*, proliferation and formation of a biofilm is an excellent survival strategy that is employed after relatively weak initial adhesion. The expression of FnBPs is critical for valvular colonization since they assist with the initial adherence to the valves, control endothelial cell invasion, and aid in shielding *S. aureus* from the immune system.^10^

### 4.5 Prosthetic valve IE

Prosthetic valve IE has been recognized as the most severe form of IE. Here we primarily provide information concerning the mechanism underlying IE associated with the prosthetic aortic valve. High turbulent shear stress exists in the vicinity of mechanical aortic heart valves, which promotes platelet activation and aggregation. The high stress increases the chances for cell damage, while regions of flow stagnation and flow separation promote the deposition of damaged cell elements, which leads to NBTE.^8^ Aortic regurgitation caused by an incomplete seal around the aortic valve leaflets can explain the abnormal high turbulent shear stress near the prosthetic valve.^11^ Also, incomplete valvular seals create suitable spaces for thrombus formation, and the implanted cardiac device itself can serve as a nidus for infection.^12^

Because microbial surface components recognize adhesive matrix molecules, methicillin‐resistant *Staphylococcus aureus* (MRSA) can colonize the fibrin‐platelet matrices of NBTE and even adhere to normal or minimally injured endothelium. The presence of cardiac prostheses is conducive to the adhesion of MRSA to the valve via biofilm formation, which facilitates prosthetic valve IE.

### 4.6 Right‐sided IE

Right‐sided IE is strongly associated with IV drug abuse, although cardiac implanted electronic device infections, indwelling lines, and uncorrected congenital heart disease also are risk factors.^13^ Repeat IV drug abusers tend to have cumulative subclinical endothelial injuries due to multiple inoculations of small bacterial loads. Also, certain substances in IV drugs can cause direct endothelial damage leading to vegetation formation. Addictive drugs, such as cocaine, can induce pulmonary hypertension through sympathomimetic actions, resulting in increased pressure gradients and turbulence, which increases the valve's vulnerability to pathogens.^14^ Moreover, the proposed pathogenesis also involves immune complex formation and deposition mainly on the tricuspid valve due to antibody‐inducing antigenic substances present in IV drugs.^14^ Intrinsic differences in the valvular endothelium, pressure gradients and jet velocities across the valves, wall stress, and the oxygen content in the right and left sides of the heart all have been proposed to explain the lower incidence of right‐sided IE.^15^

### 4.7 Embolism complications

The vegetations itself and the bacterial toxins that are produced can cause irreversible valvular damage, which manifests as valve insufficiency or regurgitation on echocardiograms. In the acute phase of IE, vegetation particles enter the blood circulation, causing vascular embolism and local vascular inflammation. The bacteria‐carrying particles cause systemic EE primarily in left‐sided IE patients, while particles from the right side of the heart cause the majority of pulmonary embolisms.^15^

A mycotic aneurysm is a rare embolic phenomenon of IE.^5^ Mycotic aneurysms most frequently occur in the aorta, visceral arteries, and cerebral arteries. Septic emboli are thought to be the precipitating event in the development of a mycotic aneurysm. Hematogenous seeding from septic emboli and endocarditis can cause infections of vessel walls resulting in aneurysmal dilatations of the blood vessels.

## 5 PREDICTORS FOR EE

Accurate prediction of embolization is critical in the early identification and treatment of risky and potentially embolic lesions in patients with IE. Considering the guideline recommendations and the number and sample size of previous studies, predictors are divided into accepted or possible groups (Tables 1 and 2).

**TABLE 1:** Accepted predictors for embolic events (EE)

Characteristics	Study type	Years of study	Sample size	RR or OR (95%CI)	*p*
Vegetation >10 mm^16^	Meta‐analysis	1983–2016	6646	2.28 (1.71–3.05)	<.001
Vegetation mobility^18^	Meta‐analysis	1994–2018	590	2.23 (1.09–4.59)	.03
Vegetations on mitral valve (vs. aortic)^18^	Meta‐analysis	1994–2018	5718	1.24 (1.11–1.37)	<.001
Vegetations on the right side^20^	Retrospective	2004–2011	1456	3.9 (2–7.7)	<.0001
CRP (>40 mg/L)^24^	Prospective	2012–2015	178	7.42 (2.06–26.72)	.002
*Staphylococcus aureus* infection^18^	Meta‐analysis	1994–2018	5591	1.64 (1.45–1.86)	<.001
ER calculator^39^	Retrospective	2010–2018	52	5.12(0.98–24.4)	.037

Abbreviations: CRP, C‐reactive protein; CI, confidence interval; EE, embolic events; ER‐calculator, Embolic Risk (ER) French Calculator; OR, odds ratio; RR, risk ratio.

**TABLE 2:** Possible predictors for EE

Characteristics	Study type	Years of study	Sample size	EE incidence (%) OR or RR	*p*
Vegetation shape^21^	Prospective	2010–2018	826	Globular versus no globular: 33% versus 0	.002
Late definitive echocardiography (≥4 days)^22^	Retrospective	2015–2017	151	Late (≥4 days) versus Early (<4 days): 40% versus 14%	.043
MPV > 8.6 fL^25^	Retrospective	2010–2012	111	MPV > 8.6 fL versus MPV ≤8.6 fL: 32% versus 11%	.01
Anti‐β2GPI IgM^a^ (+)^27^	Prospective	2007–2012	186	anti‐β2GPI IgM (+) versus anti‐β2GPI IgM (−): 75% versus 41.6%	.077
NLR^b^ > 3.045^26^	Retrospective	2011–2016	142	—	—
DD^c^ > 3393 ng/dl^28^	Prospective	2016–2018	173	—	—
Elevated troponin I^29^	Prospective	—	26	Elevated TnI versus Normal TnI: 11.54% versus 3.85%	.03
MMP‐9 > 167 ng/ml^30^	Prospective	2005–2008	145	OR 3.45, 95%CI 1.47–8.08	.006
*Streptococcus bovis* ^32^	Retrospective	1993–2001	315	*S. bovis* versus other Streptococci: 55% versus 18%	<.001
Fungi^33^ ^d^	Systemic review	Day of last search was May 20, 2020	72	—	—
*Staphylococcus spp*.^18^	Meta‐analysis	1994–2018	757	RR 1.29, 95%CI 1–1.67	.05
Young age^35^	Prospective	1995–2000	94	OR 0.39, 95%CI 0.21–0.76	.006
First episode of IE^36^	Prospective	2006–2015	1335	First episode of IE versus reinfection: 25.6% versus 14.9%	0.026
Skin manifestations^37^	Prospective	2008	497	Skin manifestations (≥1) versus none: (1)32.8% versus 18.4% for cerebral embolism; (2)51.7% versus 30.1% for extra cerebral embolism	(1) .01 (2) .001
CA^38^	Retrospective	2002–2002	82	OR 5.198, 95%CI 1.086–24.867	.039
qSOFA score ≥ 2^40^	Retrospective	2006–2017	104	qSOFA score ≥ 2 versus qSOFA score < 2: 66.7% versus 32.4%	.049

Abbreviations: β2GPI, β2‐glycoprotein I; CA, conduction abnormality; CI, confidence interval; DD, D‐dimer; EE, embolic events; MIC, minimum inhibitory concentration; MMP, matrix metalloproteinases; MPV, mean platelet volume; NLR, neutrophil‐to‐lymphocyte ratio; OR, odds ratio; qSOFA, quick Sepsis‐related Organ Failure Assessment; RR, risk ratio; TnI, troponin I.

^a^ Anti‐β2GPI IgM (+) for predicting EE: OR (95% CI): 3.45 (1.47–8.08); *p*: .0045.^27^

^b^ NLR > 3.045 for predicting EE: sensitivity 73.3%, specificity 51.9%, area under the curve 0.611, 95% CI 0.516–0.707; *p* = .028.^26^

^c^ DD > 3393 ng/dl for predicting ischemic stroke: sensitivity 78%, specificity 83%, area under the curve 0.856, 95% CI 0.77–0.92; *p* < .001.^28^

^d^ Incidence of EE in fungal endocarditis: 62.5% in patients with kidney transplantation; 63.6% in patients with liver transplantation; 84.2% in patients with heart or lung transplantation.^33^

### 5.1 Accepted imaging characteristics

#### 5.1.1 Vegetation size

Several large and contemporary studies have shown that larger vegetation size is associated with increased rates of EE, including pulmonary embolism. A meta‐analysis examined 21 unique studies published from 1983 to 2016 and concluded that cases with vegetation >10 mm in length exhibited a greater probability for the occurrence of EE.^16^

Recently, advances in real‐time three‐dimensional transesophageal echocardiography (RT3DTEE) have led to a more accurate characterization of vegetation compared to two‐dimensional transesophageal echocardiography (2DTEE). When RT3DTEE was used to measure vegetation size and area, the vegetations were more extensive than when assessed by 2DTEE. Also, RT3DTEE exhibited better embolization prediction performance than 2DTEE. The best cutoff points related to increased risk for EE during infection when using RT3DTEE and 2DTEE assessments were the maximum length of vegetation ≥16.4 and ≥9.5 mm, respectively.^17^

#### 5.1.2 Vegetation mobility

The incidence of EE was significantly higher in patients exhibiting mobile vegetations. It is not surprising that mobile vegetation can lead to a higher risk of EE, which has been proven by multiple studies over the past several years.^18^

#### 5.1.3 Vegetation location

A meta‐analysis reviewing 23 studies proved that a higher EE risk existed in IE patients with vegetations on the mitral valve versus the aortic valve.^18^ Another study noted that vegetations attached to the anterior mitral leaflet were associated with a higher incidence of embolism.^19^ The authors proposed that the anterior leaflet, which is the larger of the two mitral leaflets, can destabilize attached vegetations due to more rapid and forceful movement during valve closure.^19^ Furthermore, compared with the more “aggressive” *S. aureus*, *Streptococcus viridans* is associated with a higher incidence of mitral endocarditis. Also, the more gradual progression of infection seen with can result in larger vegetations before symptoms appear. Increased EE, especially pulmonary embolism, is an outstanding feature of right‐sided IE. In an analysis of 1456 episodes of IE, right‐side localization was one of the independent predictors of EE.^20^

### 5.2 Possible imaging characteristics

#### 5.2.1 Vegetation shape

Vegetation shape was a relevant factor that predicted outcomes in patients undergoing transvenous lead extraction. The authors defined globular vegetation as having a difference of <30% between the length and width as recorded on TEE.^21^ They reported that patients with globular vegetations demonstrated significantly higher mortality due to pulmonary embolism compared to the nonglobular group.^21^

#### 5.2.2 Time to definitive echocardiography

Many IE patients cannot be identified by an echocardiogram within a reasonable time. One study that used 4 days from admission as the cutoff point divided patients into early and late groups based on the time needed to obtain a diagnostic echocardiogram.^22^ This study revealed that a longer time to obtain definitive echocardiography was strongly associated with EE. This observation suggests that patients suspected of undergoing IE clinically but have not yet shown echocardiographic results may need close observation, and appropriate measures should be taken to prevent EE complications.

#### 5.2.3 Alternative imaging findings

Recently,^18^ F‐FDG PET has presented potential value in providing predictive information about the risk of complications associated with infections. In a study including patients with prosthetic valve endocarditis and those with native valve endocarditis, the presence of a high level of local inflammation as assessed by^18^ F‐FDG PET/CT was independently associated with a high risk of new‐onset EE.^23^

### 5.3 Accepted biomarkers

#### 5.3.1 C‐reactive protein

Serum C‐reactive protein (CRP), which is produced by the liver, is an important inflammatory biomarker. An elevated CRP level (CRP > 40 mg/L) is an independent predictor of EE.^24^ CRP activates the coagulation cascade by inducing the production of TF that modify the platelet function within the vegetation causing it to become more friable. The increased friability might explain the association of elevated CRP with EE in IE patients.

### 5.4 Possible biomarkers

#### 5.4.1 Mean platelet volume

Both endothelial damage and specific pathogens can initiate platelet activation in IE progression. Activated platelet surface receptors, such as the FcγRIIa receptor and GPIIb/IIIa, play a significant role in the process of bacteria binding to platelets. Therefore, mean platelet volume (MPV), which is associated with platelet function and activation, has achieved increased attention due to its predictive potential for the occurrence of embolism complications in IE patients. Increased MPV has been detected in patients who experienced EE compared to those who did not experience EE.^25^ Also, MPV greater than 8.6 fL has proven to be a strong independent predictor of EE.^25^ Thus, to some extent, antiplatelet therapy might reduce the risk of IE patients experiencing embolization. However, whether antiplatelet therapy is needed and effective for all IE patients is still controversial.

#### 5.4.2 Neutrophil‐to‐lymphocyte ratio

The neutrophil‐to‐lymphocyte ratio (NLR) can predict the severity and extent of cardiovascular disease. The NLR can be obtained immediately from a complete blood count. A retrospective study reported that an NLR above 3.045 measured on admission, exhibited a 73.3% sensitivity and a 51.9% specificity for predicting embolization.^26^ Neutrophils and lymphocytes are of considerable importance in the inflammatory reaction associated with infection. While the NLR can provide considerable information concerning disease severity compared to conventional biomarkers, the potential association of NLR with EE needs additional exploration.

#### 5.4.3 Anti‐β2‐glycoprotein I antibodies

Anti‐β2‐glycoprotein I (β2GPI) antibodies have been reported to enhance activation of the coagulation system and platelets, which increases the risk for thrombosis and vegetation development in patients with IE. For individuals diagnosed with IE, EE occurred more frequently over time among patients expressing anti‐β2GPI IgM, while patients who exhibited both anti‐β2GPI IgM and anti‐cardiolipin (aCL) IgM had a higher risk for cerebral embolism.^27^

#### 5.4.4 D‐dimer

D‐dimer (DD) is a cross‐linked dimer resulting from the degradation of fibrin. DD is used as a biomarker to indicate serum coagulation levels, fibrin turnover, and the autoimmune inflammatory response. Since fibrin primarily results from activation of the coagulation cascade, which commonly occurs in IE‐related tissue destruction, DD levels may be valuable in predicting EE risk. An elevated DD level might be a meaningful predictor for embolism as a consequence of increased fibrin turnover. In a recent study, D‐dimer levels ≥3393 μg/L were significantly associated with the incidence of ischemic stroke in patients with IE.^28^

#### 5.4.5 Troponin I

Troponin I (TnI) is an extremely sensitive biochemical marker for myocardial injury. TnI is frequently elevated in IE due to the presence of microbial infection and inflammation. There were statistically significant associations between elevated TnI levels and lethal complications, including central nervous system (CNS) events and major arterial embolism.^29^ In that study, the prevalence of increased TnI in IE patients with embolism might be due to coronary septic embolization with subsequent myocardial injury and the release of TnI. However, severe infection or sepsis also can cause an elevated TnI. Therefore, the association between TnI and embolism needs additional investigation.

#### 5.4.6 Matrix metalloproteinases

Matrix metalloproteinases (MMPs) are members of a family of highly homologous zinc‐dependent endopeptidases that degrade collagen and other proteins in the extracellular matrix. Due to their ability to degrade extracellular matrix components, MMPs can destroy the fragile link between vegetation and the infected cardiac tissues to which they are attached. Therefore, elevated serum levels of MMPs might be used to predict the occurrence of EE. It has been noted in a study that patients with circulating MMP‐9 values above 167 ng/ml were more likely to experience new EE.^30^

### 5.5 Accepted microbiological characteristics

#### 5.5.1 *Staphylococcus aureus*

*S. aureus* is a major pathogenic microorganism that is associated with IE. *S. aureus* infection is positively correlated with the formation of emboli and mortality due to specific *S. aureus* virulence factors and its ability to elicit extensive tissue destruction. However, isolates of MRSA have been considered to be a negative predictor of EE. One possible explanation was that methicillin resistance conferred by the *mecA* gene altered the functional surface expression of fibrinogen and fibronectin adhesins. Furthermore, the specific clonal complex, CC30, and absence of the plasmid‐borne enterotoxin‐encoding genes, *sed*, *sej*, and *ser*, were strongly associated with embolism.^31^

### 5.6 Possible microbiological characteristics

#### 5.6.1 Other microorganisms

Pergola et al. found a higher occurrence of EE in *Streptococcus bovis* infections compared to other pathogens.^32^ A recent systematic review of fungal endocarditis also reported a high embolic rate in patients infected by fungi.^33^ Another study showed that infection with *Staphylococcus spp*. also increased the risk of EE.^18^

### 5.7 Accepted clinical characteristics

#### 5.7.1 Time‐dependent factors

The majority of IE patients experience their first EE before or on the day of hospital admission, and before or on the day of echocardiography.^34^ The study showed that echocardiographic characteristics were time‐sensitive predictors of embolisms.^34^ It is plausible that EE might reinforce patients' demand to obtain medical care or hospital admission. The incidence of embolism peaked around the time when antimicrobial treatment was initiated.^4^ After the first few days of antibiotic therapy initiated, the frequency of EE decreased over time.^3^ Antimicrobial therapy can reduce vegetation size or volume and influence vegetation stability dramatically. In the early phase of antibiotic treatment, IE patients with unstable vegetations are likely to experience EE complications. Subsequently, after the infection and inflammation in the IE patients are under control, EE incidence decreases over time.

#### 5.7.2 Previous EE

A previous embolism is a known risk factor for new emboli, which probably reflects some unknown patient factors associated with the risk of embolization.^3^

### 5.8 Possible clinical characteristics

#### 5.8.1 Age

Younger patients exhibited more frequent occurrences of systemic EE. The authors hypothesized that younger IE patients might react more vigorously than older patients to an inflammatory stimulus, such as bacteremia, predisposing younger patients to embolism.^35^

#### 5.8.2 The first episode of IE

Recurrent IE has been associated with a lower risk of embolic complications, especially CNS embolism and systemic embolism. However, increased mortality in high‐risk patients during the first episode might predispose a “low‐risk” population to be more vulnerable to future reinfections.^36^

#### 5.8.3 Skin manifestations

According to one study, patients with skin manifestations (Osler's nodes, Janeway lesions, purpura, and splinter and conjunctival hemorrhages) presented a higher rate of IE‐related cerebral emboli. Janeway lesions also were associated with more extracerebral emboli. Consequently, the presence of these cutaneous lesions might indicate an active embolic process that is responsible for systemic complications.^37^

#### 5.8.4 Conduction abnormalities

Patients with cardiac conduction abnormalities exhibited a significantly higher risk of experiencing in‐hospital EE compared to patients without such abnormalities.^38^ However, an appropriate explanation for the association between conduction abnormalities and a higher risk of EE is unknown.

### 5.9 Accepted risk calculator

#### 5.9.1 The Embolic Risk French Calculator

The ER French Calculator utilizes six parameters, including age, presence of diabetes, atrial fibrillation, previous EE, vegetation length, and *S. aureus* infection. The high‐risk designation on the ER French Calculator (defined as a probability greater than 8% on the 28th day) predicted EE independently of other candidate predictors in patients with IE.^39^

### 5.10 Possible risk calculator

#### 5.10.1 Quick Sepsis‐related Organ Failure Assessment (qSOFA) scores

qSOFA scores are classified into four grades, using a scale of 0, 1, 2, and 3. Three indicators are considered, including systolic hypotension (≤100 mmHg), tachypnea (≥22/min), and altered mentation (Glasgow Coma Scale <15). One point for each parameter is added to the calculation. A high qSOFA score (≥2) was significantly associated with adverse in‐hospital events, including EE in IE patients.^40^

## 6 CONCLUSIONS

Our review presents several novel findings on the pathogenesis of IE and its embolization complications. Among the abovementioned predictors of EE discussed in this review, the predictive value of echocardiographic characteristics is the most powerful.

Easily obtained blood biomarkers also have promising prospects as predictors of EE. However, previous studies only focused on the predictive value of single biomarkers and lacked evidence concerning the efficacy of combinations of multiple markers. According to the pathogenesis of IE, we determined that the occurrence of IE and its complications resulted from the interaction of autoimmunity, alterations in coagulation systems, and pathogens. Therefore, combining biomarkers that provide a range of information might produce more comprehensive prediction values for EE complications in patients with IE.

The verification of other predictors needs further clinical investigation with larger sample sizes. Although the ER French Calculator currently is being used, it would be beneficial to design a new calculator with higher accuracy and sensitivity, which utilizes additional independent predictors of EE in IE.

Future research should focus on how to transform a more detailed understanding of pathogenesis into new methods of preventing embolism in IE patients. The exploration of additional potential predictors and clarifying the influence of different predictors in early surgical planning are urgently needed. Most importantly, the early diagnosis of IE before visible vegetation is formed and better clarity concerning the use of antibiotics to prevent EE are urgently needed.

## CONFLICT OF INTEREST

The authors declare no potential conflict of interest.

## AUTHOR CONTRIBUTIONS

Wangling Hu: conceptualization and preparation of the original draft. Xindi Wang: preparation of the original draft. Guanhua Su: review and editing of the writing and funding acquisition.

## Data Availability

The data used to support the findings of this study are available from the corresponding author upon request.
